# Groundwater irrigation reduces overall poverty but increases socioeconomic vulnerability in a semiarid region of southern India

**DOI:** 10.1038/s41598-022-12814-0

**Published:** 2022-05-25

**Authors:** Chloé Fischer, Claire Aubron, Aurélie Trouvé, Muddu Sekhar, Laurent Ruiz

**Affiliations:** 1 SELMET, Université de Montpellier, CIRAD, INRAE, Institut Agro, Montpellier, France; 2 UMR Prodig, AgroParisTech, Paris, France; 3 Civil Engineering Department, Indian Institute of Science, Bangalore, India; 4 Indo-French Cell for Water Sciences, ICWaR, Indian Institute of Science, Bangalore, India; 5 UMR GET, Université de Toulouse, CNRS, IRD, UPS, CNES, Toulouse, France; 6 UMR SAS, INRAE, Institut Agro, Rennes, France

**Keywords:** Environmental economics, Sustainability

## Abstract

The development of irrigation is generally considered an efficient way to reduce poverty in rural areas, although its impact on the inequality between farmers is more debated. In fact, assessing the impact of water management on different categories of farmers requires resituating it within the different dimensions of the local socio-technical context. We tested this hypothesis in a semi-arid area in Karnataka, South India, where groundwater irrigation was introduced five decades ago. Using the conceptual framework of comparative agriculture, based on farmers’ interviews, we built a farm typology, traced the trajectories of farm types over the last decades and assessed their current technical and economic performances. Our results show that the differentiation of farm trajectories since the 1950s has been linked with the development of groundwater irrigation, interplaying with their initial assets, and the evolution of the national and local contexts. We highlight the mechanisms by which irrigation indeed reduces poverty but engenders fragilities, particularly for poor households, whose situation was aggravated by the depletion of water resources over the last two decades. Finally, this extensive understanding of the agrarian context allowed us to formulate and assess the potential of different ways forward, including irrigation technology, change in cropping or livestock systems, land tenure, and value added distribution. As such, this analysis would be of major interest to policy makers involved in reforming the agricultural context for better agricultural water management.

## Introduction

The links between irrigation and poverty are the subject of a vast literature that converges towards the idea that the development of irrigation constitutes an efficient means of fighting poverty^1–4^. Whether it be through an increase in food production, a rise in farmers’ incomes or the creation of jobs in the farm sector and other associated sectors, irrigation improves the economic situation of rural households^4–8^. This global conclusion is nonetheless nuanced by the works dealing with the issue of equity, which show that the development of irrigation can go hand in hand with a reinforcement of inequalities^9–13^. These inequalities are partially geographical^14^—the regions offering better possibilities for irrigation benefit more from the positive effects of its development—but are also due to the contrast between households within a same region^15^. Households’ access to land^4,16,17^, the capital they possess^10,11,18,19^, their precocious or late investment in irrigation^20,21^, the sources of irrigation they make use of^9,12,22^, the social relationships they are part of and that structure water governance^23,24^ translate into differentiated effects of irrigation development between households. Another emerging question is how the depletion of water resources observed in certain irrigated agriculture regions affects equity^10,11,19–21,25–27^: are poor households more vulnerable than better-off households? If so, what are the contributory mechanisms?.

Apart from the question of the differentiated effects of the depletion of water resources on households, the subject of the links between irrigation and poverty is not new. However, the works that articulate these different dimensions and assess the effects of the development of irrigation on different social categories, remain relatively rare^28^. Such studies carried out at a regional or micro-regional scale, which includes field surveys, are nonetheless necessary to allow us to understand how water management is embedded in a local socio-technical context and to take this into account in policy formulation and irrigation development actions^29–31^. By studying the trajectories of farms in the region over time, it is for example possible to identify the mechanisms that give the households who first developed irrigated cropping an advantage over those who turned to irrigation later and to assess the resulting income differences that exist today. Such studies also enable an objectivisation of the role the capital assets agricultural households possess play in their ability to take advantage of irrigation, and to assess the effect of debt on their income. The work and jobs generated by the development of irrigation are a third field of investigation that deserves to be explored using these integrated approaches: what is the scale of the labour requirement induced by irrigated agriculture in comparison to rainfed agriculture? Is the increase equivalent for all irrigated cropping systems? Who does the work and what kind of value sharing exists when the workers are paid labourers? A combined approach to these different dimensions (farm trajectories, capital assets, effects on work and employment) seems to be particularly useful when it comes to understanding the impact of the development of irrigation on agricultural households, and assessing the extent to which it reduces poverty and ensures equity.

In this article, we provide an integrated analysis of the development of irrigation in a semi-arid zone in the State of Karnataka in South India. As is the case in many places in India, this development has taken place through deep borewell drilling, often considered more accessible to smallholders than vast irrigation projects^32,33^. We based our analysis on a field study making use of the conceptual framework of comparative agriculture, which allowed us to trace the trajectories of different types of farms over the last decades, and to assess their current technical and economic performances. The overall results highlight the mechanisms by which irrigation indeed reduces poverty but engenders fragilities, particularly for poor households, whose situation was aggravated by the depletion of water resources over the last two decades.

## Methodology

### Study area

**Figure 1:**
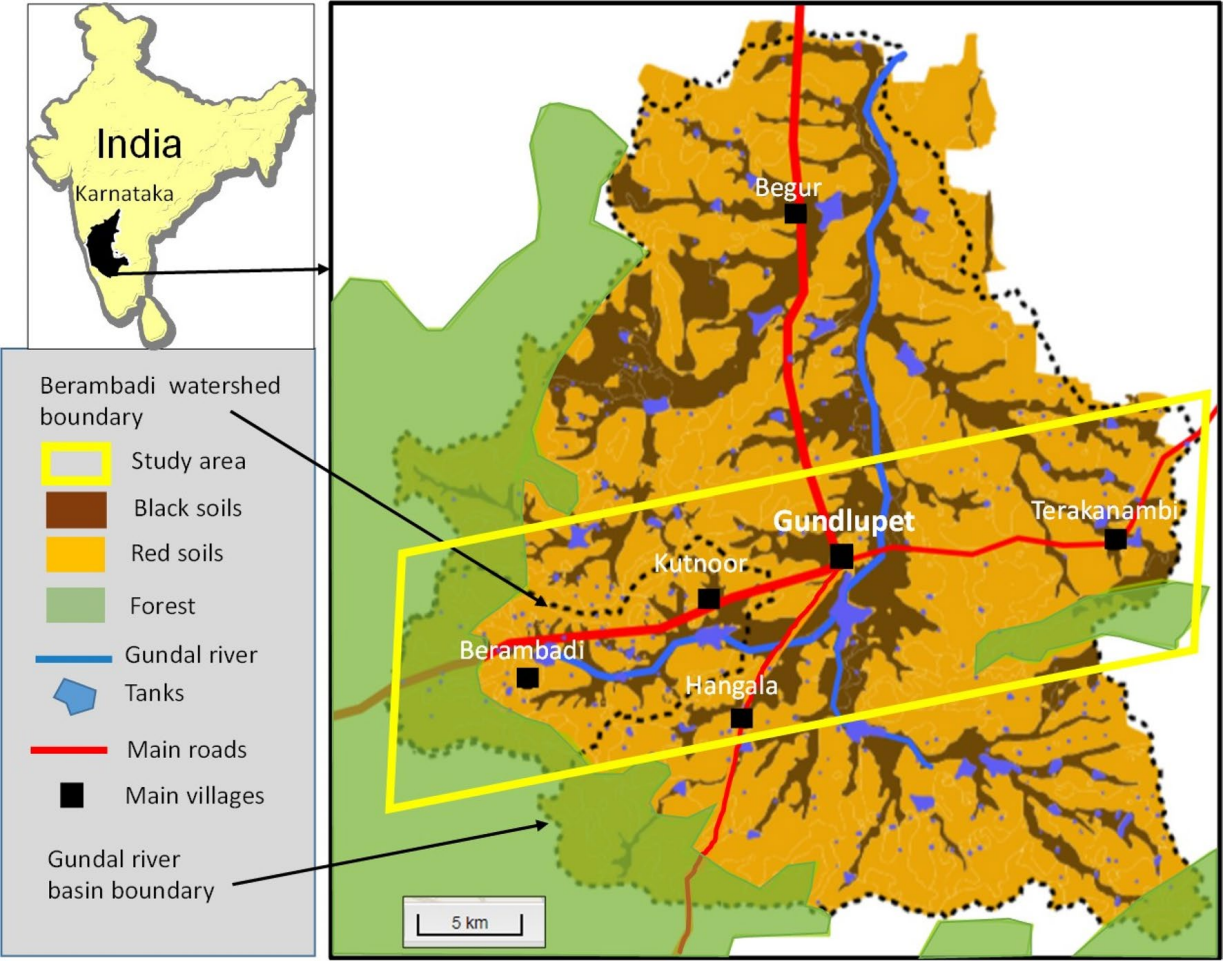
Location of the study area in Gundlupet Taluk, South Karnataka, India. The Gundal flows northward, and the river basin boundary closely follows that of the Taluk. The western part of the study area comprises the Berambadi experimental watershed (84 km^2^). We generated the map with 4georchestra version 2.17 (https://geosas.fr/portails/?portail=aicha).

The study area (Fig. 1) located in the South of the Deccan plateau, in Karnataka state, Chamarajanagar district, Gundlupet taluk, India. According to the 2011 Census of India, the total population of taluk was 223 070 and the two commonly most disadvantaged groups represented respectively 19.3% (scheduled caste) and 12.9% (scheduled tribe) of the total population. The working population was composed of 37% of agricultural labourers, 28% of cultivators, 21% of household industries and other workers and 14% of marginal workers. At the district level, according to the NITI Aayog baseline report^34^, 18,91% of the population was poor and the multidimensional poverty index was 0,079, figures that are rather high for Karnataka (headcount ratio 13,16% and MPI 0,056) but low for India (headcount ratio 25,01% and MPI 0,118).

The study area covers about 300 km^2^, and is centred around Gundlupet town (longitude 76.69, latitude 11.81, in WGS 84). It belongs to the Kabini Critical Zone Observatory^35^ and to the Environmental Research Observatory M-TROPICS^36^ (https://mtropics.obsmip.fr), which is part of the Research infrastructure OZCAR^37^ (Critical Zone Observatories: Research and Application, http://www.ozcar-ri.org/ozcar/). It is part of a steep climosequence caused by the Western Ghats rain shadow^38^, with the average annual rainfall decreasing from about 900 mm in the West to 600 mm in the East (Fig. 1). The area is relatively flat (altitude above sea level between 900 m in the West to 700 m in the Gundal valley) with a bedrock composed of a Precambrian gneiss covered by an immature and heterogeneous saprolite layer^39^. In such a crystalline context, aquifers have low water storage and transmissivity, both declining with depth^40^. The soil cover is mainly composed of a red soil–black soil system, with shallow red soils (Ferralsols and Chromic Luvisols) on the hillslopes and deep black soils (Vertisols and Vertic intergrades) in the valley bottoms^41^.

The West and South of the area are covered by a dry deciduous forest, which is part of the Bandipur National Park, the most protected zone (“core area”) of the Nilgiri Biosphere Reserve. The rest of the area is dominated by agriculture. The monsoon dynamics drive three main cropping seasons: the Kharif season (from May to August, receiving about half of the yearly rains, comprising early rains from convective storms followed by the South-West monsoon), the Rabi season (from September to December, receiving the other half of the rains from the North-East monsoon) and the Dry season (also called “summer season”, from January to April, with very rare rainy events implying that only irrigated crops are possible). The Potential Evapotranspiration (PET) is about 1200 mm/y and the aridity Index^42^ (AI = P/PET) ranges from about 0.75 in the West (sub-humid) to 0.5 in the East (semi-arid). This makes the region very vulnerable to negative water balances, leading to groundwater depletion when the area under groundwater irrigation increases. The major effect of groundwater pumping on the progressive groundwater resource depletion observed since the 1990s was demonstrated at the scale of the Gundal river basin^43^. Remote sensing approaches enabled a reconstruction of the evolution of the spatial distribution of groundwater irrigation since the 1990s for the Berambadi watershed, showing that while it started in the valley bottoms and in the East, it later developed on the hillslopes and towards the West of the watershed^44^. To date, even though many more borewells have been drilled in the East, many of them have stopped yielding water there and the average number of functioning borewells per farm is uniform across the watershed^45^. Excessive groundwater pumping impacted not only groundwater availability^35^ but also its quality, with widespread contamination by nitrates^46^ and incipient salinization^47^.

### Agrarian diagnosis

#### Conceptual framework

Agrarian diagnosis is an approach to farming system dynamics and diversity at the micro-regional scale. It is part of the *comparative agriculture* framework designed to study and compare agricultural development processes in different parts of the world and historical periods^48^. The combination of social and biotechnical sciences that view agriculture within a single framework is one of the main specificities of comparative agriculture. This is what enables an analysis at different scales, as well as the articulation of scales. A first concept, the *agrarian system*, is used at the broader regional or micro-regional scale^49^. Defined as “the theoretical expression of a historically constituted and geographically localized type of agriculture”^50^, the agrarian system is composed of two interacting elements: (i) the farmed ecosystem, encompassing one or several ecosystems and the associated farming practices and (ii) the social relations and rules organizing access to resources, production and trade. An agrarian system includes a limited number of so-called *production systems*. This second concept—the production system—refers to “a group of farms with the same range of resources (same area, same level of equipment, same workforce) in similar socio-economic contexts, with a similar crops and livestock mix”^51^. Highlighting the differentiation process of these groups of farms, and thus the current diversity of production systems within the agrarian system, is an essential step in the research in comparative agriculture. Finally, a third category of concepts taken from agronomy and animal sciences, respectively the *cropping system* and the *livestock farming system,* makes it possible to tackle practices implemented by the farmers at plot and herd scale. Far from stable, the cropping systems, livestock farming systems and production systems as well as the agrarian system of a given region are constantly evolving, owing to several internal and external factors (e.g. local environmental change, migration, conflicts, availability of new equipment, rise or fall in market prices, etc.)^48,52^. Paying attention to the identification of these factors of change and the analysis of their systemic impacts at different scales ensures a dynamic perspective on agriculture.

The approach is largely based on interviews. In this study, all interviewees gave informed consent for participation. No personal information is disclosed in this publication. The research protocol wasn’t subject to formal review by an Institutional Review Board as it didn’t meet requirements for assessment (limited to health research).

#### First phase: agro-ecological zoning and reconstitution of agrarian dynamics

Agrarian diagnosis relies on intensive fieldwork, which was carried out for this study between March and August 2016. It started with landscape analysis through one-month direct observations and a review of available maps, biophysical environment data and satellite imagery aiming at identifying landscape zones that were homogeneous in terms of biophysical conditions (climate, topography, soils, vegetation, etc.) and agricultural patterns (crops, plots size and spatial organization, herds, infrastructures related to agriculture, etc.). The underlying assumption is that biophysical conditions define at least partly the range of farming practices that can be implemented. Having access to one agro-ecological zone or another is thus not neutral for farmers^53^ and contributes to explaining the diversity of farming systems.

Then, the following month (April–May 2016), 46 interviews were conducted, mostly with older farmers, combined with the consultation of available written sources on the agrarian history of the area. Here, the objective was to reconstitute the local agrarian dynamics since the 1950s and identify the main environmental, technical and socio-economic drivers of changes. Special attention was paid to the different trajectories of farms over time, and to the factors explaining such differences. The sample was built progressively: first respondents were selected randomly within each agro-ecological zone and the following chosen to cover the diversity of farm trajectories that we gradually highlighted. We identified them based on our direct observations as well as on suggestions from previous participants (snowballing)^54^.

The agro-ecological zoning of the study area and the reconstitution of its agrarian dynamics led to a first understanding of the current diversity of farms, which helped select farmers for the next round of interviews.

#### Second phase: characterization and assessment of current farming systems

The second phase of the fieldwork, spread over approximately three months (May–August 2016), consisted in observations of agricultural practices and about 90 interviews with active farmers. It aimed at characterizing more precisely the structure and the functioning of the farms in their diversity, and assessing their technical and economic results. Farm structure refers to access to land, water, equipment and labour force. Farm functioning is defined by cropping practices (choice of crops and cultivars, crop rotation and associations, calendar of technical operations for each crop, the associated work calendar and workload, yields etc.), and livestock farming practices (choice of breeds, breeding, feeding and animal health management practices, use of the products, the associated work calendar and workload, yields, etc.). It also includes material and energy flows between crop and livestock, work organization at the farm level as well as economic data regarding prices, equipment lifetime and day labourers’ wages. Observations and interviews were conducted so as to cover the diversity of farms, which had been pre-identified during the first phase and was finally translated into a typology of farms. The same methods used in first phase served to identify interlocutors fitting the criteria for the interviews (direct observations and snowballing).

Based on this in-depth understanding, we then built archetypes representing the highlighted diversity of production systems (6), cropping systems (8) and livestock farming systems (2) in the area. A production system is composed of at least one cropping system or one livestock farming system and may combine several cropping and livestock farming systems. The archetypes are a detailed qualitative as well as quantitative characterization of the structure and functioning of each identified farming system^55^. In methodological terms, they correspond neither to real farms, nor to sample values averages. They are the product of a modelling process consisting in “performing an informed reduction of the observed variations”^55^, based on an understanding of the technical and socio-economic processes operating on the ground acquired through the previous research steps. Compared with multivariate clustering procedures^56^, this method has proven successful in characterizing regional farming system heterogeneity^57^.

Finally, we assessed the economic performance of farming systems, considering two types of indicators: value added and income. *Value Added* measures the wealth creation “intrinsic to the productive process”, whereas *agricultural income* results from a further distribution of the value added^58^. We calculated the value added at the level of the cropping system, livestock farming system and production system (see Supplementary material I). To assess the economic efficiency of the productive processes, we then compared the *productivity of factors* between farming systems^58^. Land productivity and labour productivity refer respectively to the value added per surface area and per required labour force^58,59^. Finally, we assessed the agricultural income at the production system level. It is the share of the net added value that remains in the farmer’s hands, once other economic actors who contributed to providing access to productive resources in this type of farms have been remunerated (see Supplementary material I). Where applicable, we thus deducted land rental, wages paid to hired labourers and loan interest from the value added to calculate the agricultural income. When relevant, we also considered the other incomes related to agriculture such as agricultural labour wages or renting out of a tractor. At the farm level, value added and income indicators were calculated not per household but per family worker—i.e. per person who works in the farm of his/her family – to be able to compare farm types with different numbers of family workers.

## Results

**Figure 2:**
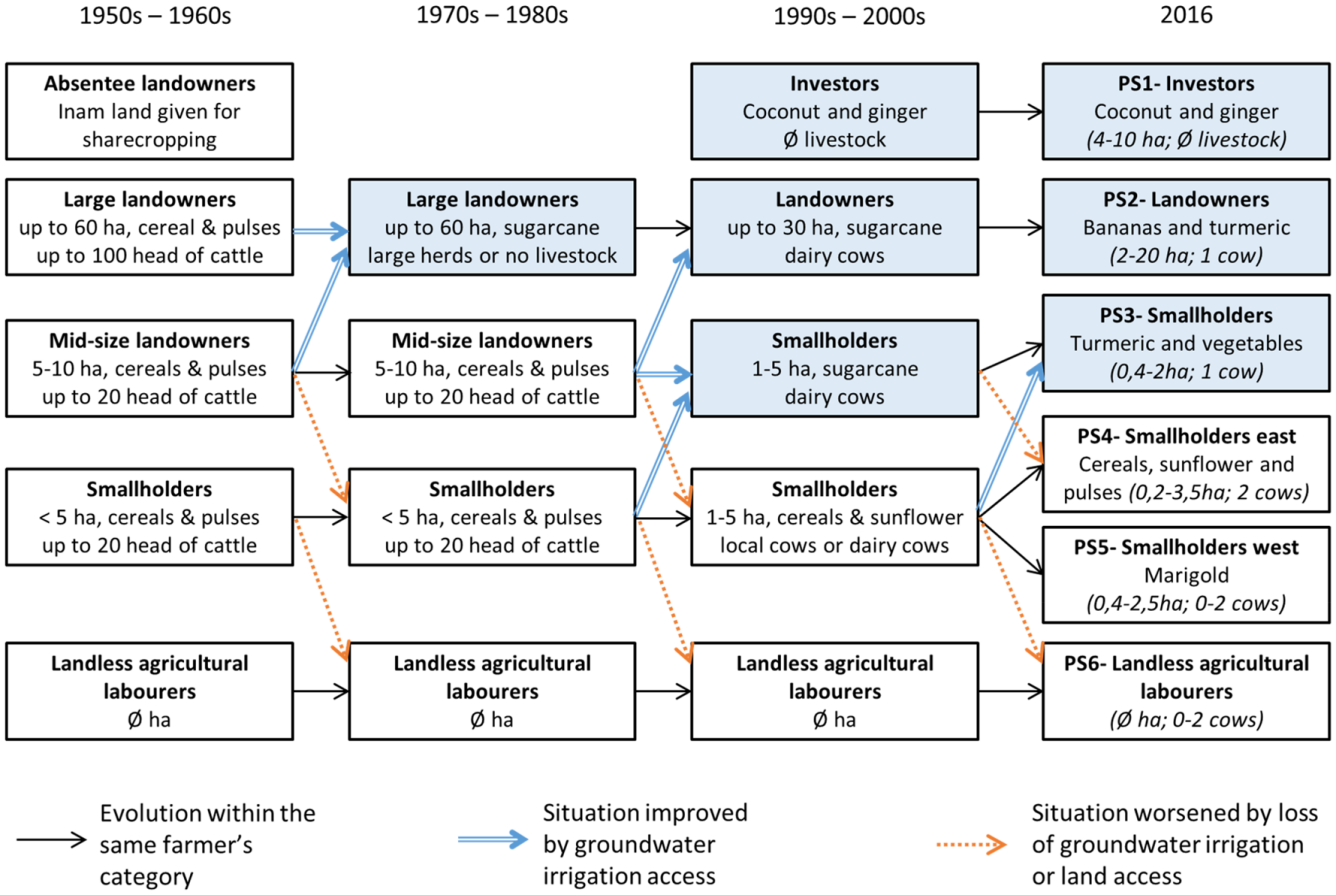
Evolution of production systems (1950s–2016). The blue boxes indicate access to groundwater irrigation.

The development of groundwater irrigation has been the main driver of farm trajectories over the past 50 years in the area, as shown by the reconstitution of the agrarian dynamics (Fig. 2), and access to groundwater irrigation remains one of the main factors of farm socio-technical differentiation.

### 1950–1960: an agrarian system unequal in terms of land and capital

In the 1950’s, all farms were engaged in crop cultivation for family self-consumption, associated with livestock activities. Most of the cultivated land was rainfed, except a few percent of the total cultivated area, which was irrigated with surface water from the traditional “tank system” allowing for two rice cycles a year. The groundwater table was very shallow and the Gundal river flowed permanently. Most of the rainfed land produced two crops a year, mainly cereals (sorghum and finger millet) in the Kharif season and pulses (horsegram) in the Rabi season while only large and medium farms also cultivated cash crops such as groundnut. Cereal surpluses and groundnut were sold in Terakanambi, the historical agricultural market located in the east of the taluk (Fig. 1). All farmers owned a herd of local cattle (from 20 to 100 head depending on the size of the land), and their main purpose was to provide manure for fertility maintenance through spatial transfers between rangeland and cultivated areas, and draught power. Cattle were kept in pens during the night and send to graze inside the forest or on common grazing lands in the daytime.

Large and medium landowners were mainly Lingayats, a community traditionally involved in farming in Karnataka. On the largest Lingayat farms, part of the land was worked under sharecropping agreements. The landowners cultivated the most fertile land in valleys while the tenants farmed the hillslopes. In addition to their family work, the landowners hired permanent as well as daily labourers to handle the work peaks such as harvests. In addition, some large agricultural estates called *Inam lands*, granted to Brahmins by royal and colonial authorities during the colonial era^60^, still existed in the 1950’s. Most of them were located in the Gundal valley and included land in the traditional tank command areas. The Brahmins were absentee owners, as they generally lived in towns or cities where they held high positions, for example in the administration, and their estates were cultivated by farmers from lower castes under sharecropping arrangements.

Smallholders were sociologically a more diverse group: people belonging to craft and service castes could also be farmers on their own land—generally distant from villages—or sharecroppers on Lingayat or inam lands. Most of the remaining population, including lower caste people, had no access to land and worked as agricultural labourers.

The agrarian reforms implemented after independence (1954 Mysore Inams abolition Act; 1961 and 1974 Land Reform Acts)^61^ had little impact on the tenure situation in the area. In fact, only the inam lands, which were officially abolished in 1955 in Karnataka, were redistributed to the tenants^61^. Lingayat farmers, who owned most of the land in the area, were little affected, and some of them even benefitted from the redistribution of inam lands, on which they were sharecroppers.

Large landowners accumulated capital not only from their farm produce, but to a large extent from the loans they provided to other local stakeholders, at a high interest rate (about 25% per year in the case of consumption loans). Landless people took loans for consumption, as well as to pay the dowry to marry their daughters. This debt system observed elsewhere in India^62,63^ also gave large landowners access to almost free labour, as people with nothing left to sell worked for the money lender until their loan was fully reimbursed. Smallholder farmers generally mortgaged their land in favour of their creditor in exchange for the loan of a sum of money, and when they were unable to repay the loans, they were forced to sell it to them. This practice, which was also described in Eastern India^64^, continues today, leading to significant land flows from small to larger farmers.

### 1970s-1980s: the development of open wells for groundwater irrigation initiated by the better-off farmers

The study area was not directly impacted by the Green Revolution (initiated in the late 1960s in India) which mainly targeted existing surface irrigation infrastructure, and the production of rice and wheat^65^. Yet, high yield seeds for a variety of crops, subsidized chemical fertilisers and pumps, became widely available. Sugarcane started to be an interesting gamble as production was largely encouraged by the Indian government and the market price for sugar tended to rise over the period. However, given the importance of the investment required, most of the farmers who could seize this opportunity were the better-off large landowners who owned land in the deep black soils in valleys (Fig. 1) particularly suitable for this crop. Benefitting from the availability of shallow groundwater, they invested in open wells for irrigation (hiring labourers for the well construction) and pumps (first diesel pumps, then electric pumps when the electricity network became available). Moreover, as no sugar factory was close enough to the study area, they had also to invest in their own jaggery mill to process their sugarcane production. The development of irrigation began in the South and East of Gundlupet, because of the larger proportion of black soils and the proximity of the Terakanambi market.

A few mid-size landowners with access to suitable black soils and sufficient land to allocate a part of it to sugarcane, also dug open wells through loans taken from large landowners. Although they often had to get their production processed in their creditors’ jaggery mill (at a cost), they could generally pay off this loan quite easily thanks to the favourable market conditions for sugar at that time. As land pressure increased in their own State, some farmers from the neighbouring State of Tamil Nadu bought land from local indebted farmers and settled in this area. They looked for affordable farm land to avoid excessive subdivision of the family farm between heirs and they were attracted by the availability of cheap labour and groundwater in Gundlupet area. They came with a significant capital and were able to invest in open wells to grow sugarcane, as well as turmeric, that they introduced into the area.

However, most of the small and mid-size landowners in the area did not have enough capital to invest in irrigation. Yet, their cropping patterns also evolved, as some began to grow hybrid varieties of rainfed crops (finger millet and sorghum) using chemical fertilisers. As the importance of subsistence agriculture decreased with the increase of subsidised food grains coming from the Indian surplus regions, they started growing rainfed cash crops such as sunflower and cotton.

The development of irrigation provided more daily job opportunities for landless labourers and small farmers, particularly in the sector where sugarcane was the most developed (South and East of Gundlupet). Indeed, cropping systems either with sugarcane or turmeric were more labour-intensive than rainfed crops and in addition, digging open wells and sugarcane processing were labour intensive activities.

The land reforms continued during this period. In particular, a land ceiling of 4 ha per household was set, and some of the large landowners’ land was redistributed to the tenants. However, the implementation of the reform was limited, and although some land was handed over to low caste farmers, the Lingayats still owned most of the best land in the area^61^. The reduction of farm size, due to population increase and the division of farmland between heirs, was partly compensated by the expansion of cultivated lands on the hillslopes, at the expense of public grazing lands. With thin and rocky soils with minimal water holding capacity, these areas have a low agricultural potential.

This reduction of the grazing land area partly explains the strong decline in livestock farming that occurred in this period. The cultivation of long cycle crops on irrigated land also reduced the surface available for grazing after harvest. Finally, access to the forest for grazing was restricted when the Bandipur national park was established as a tiger reserve in 1974. As a result, many large landowners decided to quit livestock farming and to specialize in irrigated crop production, and they partly replaced manure with chemical fertilizers. In contrast, small and mid-size rainfed farmers maintained their herds of local cattle. They continued to use draught power and produce farmyard manure for their own fields, and also started selling a part of it to farmers who no longer reared livestock.

### 1990s-2000s: the expansion of borewells for groundwater irrigation and the “golden age” of sugarcane

In the early 1990s, the introduction of submersible pump technology was a game changer, allowing a wide range of land-owning farmers (even those with little or no capital) to gain access to irrigation as in many parts of monsoonal Asia^66^. As groundwater table in the valleys decreased, open wells began to dry up. Borewells equipped with submersible pumps allowed to go deeper and were strongly incentivized by the Karnataka government, who started providing free electricity for farmers in 1992^67^. Other factors contributed to the rapid expansion of borewells, and to the “golden age” of sugarcane, a crop that became ubiquitous in the centre and east of the area. First, in 1992, a sugar factory was opened in Nanjangud, 40 km north of Gundlupet, providing advances to farmers for inputs, and a guaranteed price fixed by the government; this removed the obligation for sugarcane growers to invest in their own mills. Furthermore, the evolution of relative prices of most irrigated crops and nitrogen fertilizers (subsidised) encouraged the development of fertilizer-intensive crops^68^. Finally, the links developed between Gundlupet and the neighbouring States of Tamil Nadu and Kerala created market opportunities for crops such as turmeric and onion (grown in association), and although very volatile, the prices were high at that time.

Large landowners and Tamilian farmers were the first to adopt the submersible pump technology, as they had sufficient capital to invest without taking loans. Despite their generally smaller farms, Tamilians mastered the agricultural techniques of groundwater irrigated crops, and in addition, had connections with the Tamil market. Otherwise, the large majority of small farmers required external capital support to take this step. As a result, they either sold a part or their land or livestock or took out heavy loans at high interest rates (between 3 and 5% per month), essentially from farmers who had invested in irrigation in the previous period. Therefore, although consumption loans—and hence the associated free labour—had nearly disappeared partly thanks to the development of food rations provided by the government, large landowners continued to procure these investment loans to accumulate capital—and land, when the borrower faced bankruptcy.

The region became attractive to alien investors as cheap land was available due to farmer’s debt, labour wages were low and groundwater was available. Wealthy businessmen from Kerala bought plots of land along the main roads from different owners, to create medium to large estates, where they planted coconut trees and cultivated understorey irrigated crops, such as banana and ginger. The landowners were absentee and the farm was managed by a permanent worker who hired daily labourers.

Many smallholders did not invest in irrigation, yet several changes occurred in their rainfed cropping systems. Government incentives for oilseed crops (the “yellow revolution”)^65^ led to the expansion of sunflower to the detriment of groundnut and cotton. At the end of the 1990s, companies processing marigold into colourants settled in Gundlupet and contracted rainfed farmers in the west of the area to grow marigold. They provided advances for inputs and prices guaranteed through an annual contract. In addition to this new cash crop, the wetter climate in the west of the taluk allowed development of rainfed maize in the Rabi season at the expense of traditionally grown pulses.

The increase in population and land division was less and less compensated by the expansion of agricultural land, as available range land was shrinking. The average size of farms in all categories declined. However, as labour demand increased with the development of irrigated lands, small rainfed farmers were able to compensate the reduction of their farm size with more job opportunities, which also helped the increasing number of landless labourers to ensure their livelihoods.

Another market opportunity contributed to improving farmers’ livelihoods: within the framework of the government programme to increase milk production (the so-called “White revolution”, which started in Gujarat in the 1970s)^65^, dairy milk cooperatives were created in the 1990s in villages in the Gundlupet Taluk. Milk was collected from any volunteer farmer, regardless of the volume supplied and was paid weekly at a stable price. Services and inputs such as artificial insemination and feed concentrate were also provided. Milk production became a valued source of income for farming families. Both rainfed farmers and new irrigators replaced their local cattle breeds with more productive cross-breed dairy cows. Even some of the first irrigators who had previously abandoned livestock farming, bought Jersey or Holstein cows to produce milk. From this period onwards, the main objective of livestock farming shifted from manure and draught power to milk, for sale and self-consumption. On irrigated farms, cows were easy to feed with crop by-products and irrigated forage (sugarcane, maize and elephant grass). Rainfed farmers continued to rely mainly on pasture, complemented with cereal straws during the dry season, but as seen previously, they also began to grow fodder maize in the Rabi season. Even some landless labourers bought cows, which they fed with spontaneous grass collected on the roadsides or in the fields and supplement with purchased fodder and feed concentrate.

### From the mid-2000s onwards: the groundwater crisis

The rapid increase in the number of deep borewells during the previous period caused a dramatic decrease in groundwater table levels, first in the valleys, which had the highest density of drilling, especially in the centre and East of the study area^44^. The total investment cost could reach up to 170 000 Rs per hectare in 2016 (for a 200 m deep borewell, an electric pump and the equipment for drip irrigation). As a consequence, the Gundal river was disconnected from the aquifer and dried up completely in the early 2000s, as did most of the traditional tanks, marking the end of rice cultivation in their command areas. As the water table declined, so did the borewell yield.

In the east, as many borewells stopped yielding water, farmers tried to retain their access to irrigation by resorting to massive investments. Those who had started to grow irrigated crops in the earliest period possessed sufficient capital. They invested in deeper and deeper borewells (up to 300 m) and in newly available water saving techniques, such as sprinklers and more frequently drip irrigation^45^. The few smallholders who managed to retain their access to irrigation are now heavily indebted, while the others had no choice but to return to rainfed agriculture, often with a loan repayment burden, forcing them to sell their livestock or even to hand over their land to their creditor. In the west, where relatively shallow groundwater was still available, borewell drilling and the irrigated area continued to expand. A large number of irrigators, even the indebted smallholders, invested in drip irrigation in this area.

As observed in other parts of India^69^, the general decline in groundwater resource availability induced dramatic shifts in cropping patterns throughout the study area. Sugarcane is very water intensive, little adapted to drip irrigation and cannot be combined with shorter cycle crops which give a quicker return: it virtually disappeared from the area in a few years. On medium to large irrigated farms, it was partially replaced by banana, which is well adapted to drip irrigation and can be associated with one cycle of vegetables at the beginning of its cycle. Smallholders seldom cultivate banana, because the long duration of its cycle makes it too risky in the context of uncertain groundwater availability, and also because the price is highly volatile (ranging from 5 to 25 Rs/kg at the time of the study). Turmeric is currently the main irrigated crop on all the irrigated farms (except on investor estates). It is favoured because it has a shorter cycle (9 months, as compared to 12 for sugarcane or banana), can be associated with one cycle of onion (3 months) at the beginning of its cycle, and brings high profits when the prices are favourable. Vegetable production is gaining importance as it involves short duration crops (2 to 4 months) that provide a quick return on investment and is less risky than annual crops particularly in situations of groundwater depletion in borewells. Indeed, when groundwater is available, 3 cycles of vegetables can be grown per year. Vegetable cultivation is undertaken mostly by irrigating smallholder farmers, who manage the risk associated with price volatility by diversifying their production on smaller and smaller plots.

For rainfed farmers, there was little change in cropping patterns during this period. As the demographic trend described for the previous period continued, the size of landholdings continued to decrease—for all farm types—and was no longer balanced by an expansion of the cultivated area, as the territory had reached saturation. With the reduction of straw availability in the area, induced by the development of crops that do not provide straws (turmeric, vegetables, marigold, sunflower), it became increasingly difficult to maintain dairy cows and some of the rainfed farmers cultivating marigold in the west abandoned livestock farming. The decline in livestock farming is also linked to the increasing mechanisation of certain cropping operations, particularly ploughing. Many large landowners who had invested in tractors in the previous period became rural entrepreneurs earning additional income by renting them out as in other parts of South Asia^70^. In 2016 a tractor cost on average 600,000 Rs (8900 USD) and one-hour of tractor ploughing was charged 600 Rs (8.9 USD). Interestingly, rainfed farmers are increasingly using their services for ploughing operations in their own fields, while conversely they are paid to prepare the seedbeds for large landowners with the bullocks they still own.

Overall, the period has seen a decline in the demand for daily labourers, not only due to the reduction of the irrigated area, but also because micro-irrigation is less labour intensive than the traditional furrow technique. In addition, the new dominant crops such as banana and turmeric are less labour intensive than the former sugarcane processed in jaggery factories. Landless labourers from the villages in the east of Gundlupet commute daily to the west of the area, where the demand for daily labour is still high, but many of them now have to undertake seasonal migration, working as agricultural labourers on plantations in the neighbouring state of Kerala to supplement their incomes.

### Current production systems diversity and assessment

**Figure 3:**
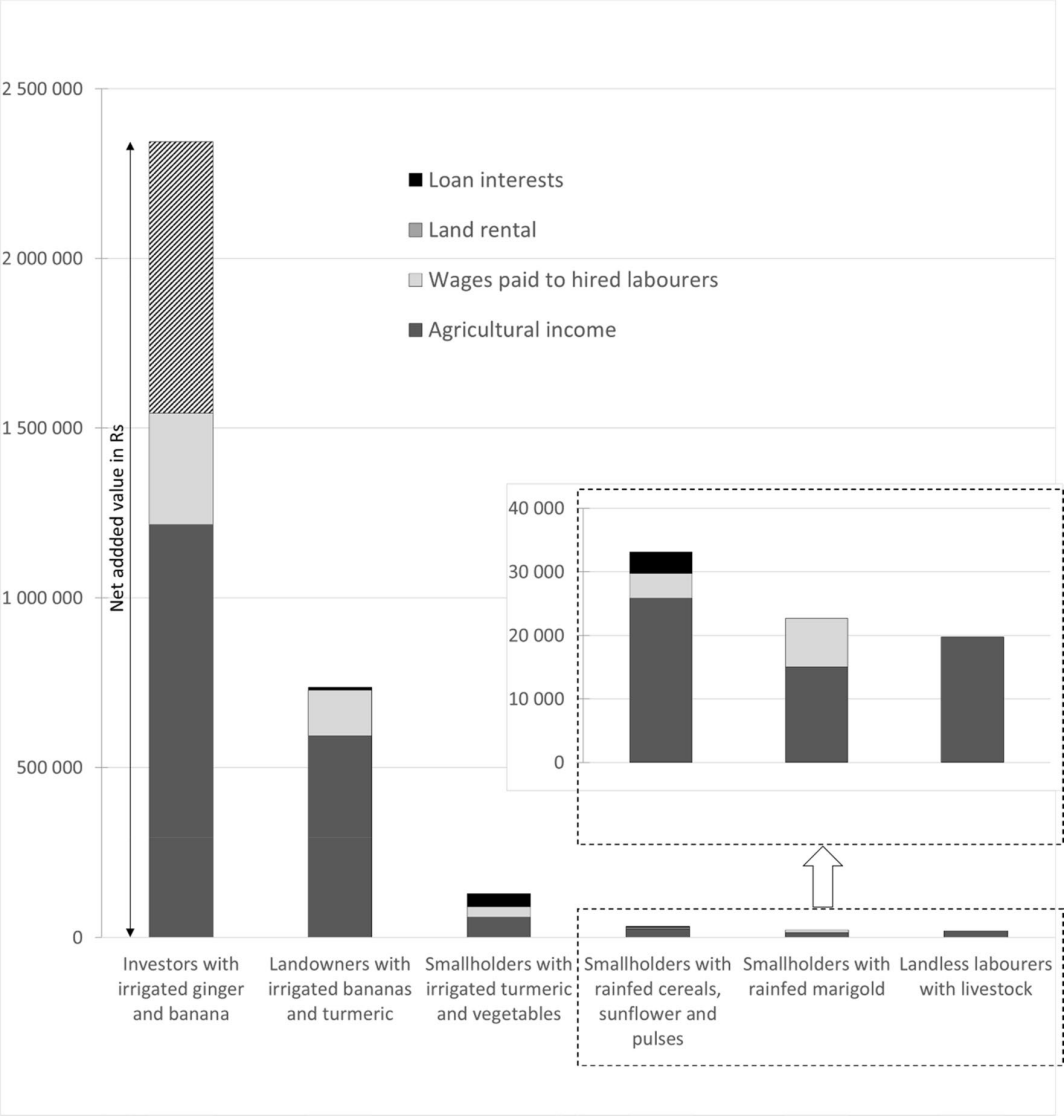
Distribution of net value added and resulting agricultural income for 6 production systems, in Indian Rupees (Rs) per family worker or per owner per year. The Y axis for the 3 production systems on the right is expanded in the inside plot to allow visualizing the distribution of the net value added. Exchange rate with US Dollar (USD) was 1 USD = 67.5 INR.

As explained in the previous sections and represented in Fig. 2, the diverse production systems that exist today result mainly from the unequal relations between the different farmers’ categories that led to inequalities in accessing land and capital, and therefore water. In this section, we consider six production systems (PS1 to PS6) representing this diversity, three of them with access to groundwater irrigation. With the exception of Keralan investors, all production systems with access to land are owners of the land, sharecropping and renting being insignificant today in Gundlupet. Table 1 compares the cropping systems (for details on the cropping and livestock farming systems, see supplementary materials part II) while Fig. 3 and Table 2 compare the production systems at the farm level.

**Table 1:** Comparison of cropping systems: use of external inputs, labour demand and productivities.

	Use of external inputs (Rs/ha/year)	Labour demand (days/ha/year)	Irrigation seasons	Yearly land productivity: Gross Value added (Rs/ha/year)	Daily labour productivity: Gross Value added (Rs/day)
Ginger associated with chilli	405 167	777	Dry season + Kharif + Rabi	786 500	1012
Vegetables and bananas on a two-year cycle	108 554	308	Dry season + Kharif + Rabi	530 000	1714
Associated turmeric	168 649	716	(late dry season) + Kharif + Rabi	480 000	674
Vegetables (3 cycles in a year)	163 333	736	Dry season + Kharif + Rabi	420 000	572
Sunflower followed by horsegram (East)	57 204	112	/	38 136	340
Sorghum followed by horsegram (East)	30 107	137	/	45 160	328
Maize followed by horsegram (West)	30 197	143	/	70 459	491
Marigold followed by horsegram (West)	67 224	269	/	67 224	250

Prices are in Indian Rupees (Rs). Exchange rate with US Dollar (USD) was 1 USD = 67.5 INR.

**Table 2:** Comparison of production systems: fixed capital, net value added and incomes.

	Fixed capital (total initial value in Rs)	Net value added (Rs/year)	Agricultural income (Rs/year)	Livestock contribution to agricultural income (%)	Other income related to agriculture (Rs/year)	Total income related to agriculture (Rs/year)
PS1- Investors with irrigated ginger and banana (1 owner; 5 ha; Ø livestock)	837 336	2 344 198	1 215 522	0	0	1 215 522
PS2- Landowners with irrigated banana and turmeric (2 workers; 3,5 ha; 1 cow)	2 296 883	736 535	593 008	4	106 272	699 280
PS3- Smallholders with irrigated turmeric and vegetables (2 workers; 0,6 ha; 1 cow)	232 132	128 549	59 924	37	0	59 924
PS4- Smallholders with rainfed cereals, sunflower and pulses (2 workers; 0,8 ha; 2 cows)	26 400	33 099	25 872	67	5 295	31 167
PS5- Smallholders with rainfed marigold (2 workers; 0,8 ha; Ø livestock)	1 400	22 722	15 053	0	18 255	33 308
PS6- Landless labourers with livestock (2 workers; Ø ha; 2 cows)	20 700	19 781	19 781	100	10 560	30 341

All indicators in Indian Rupees (Rs) per family worker or per owner. Exchange rate with US Dollar (USD) was 1 USD = 67.5 INR.

Investors from Kerala (PS1) implement cropping systems based on ginger and banana, with high land and labour productivity (Table 1). Those examined here cultivate on rented land, renting new farms every three years to reduce the risk of fungal root diseases in ginger and to cope with groundwater depletion in borewells. The rental costs have a huge impact on their economic results (34% of the value added, see Fig. 3) but the income produced by this type of farm is by far the highest of the six types studied here (Table 2).

Mid-size irrigated farm owners (PS2) have two origins: they are either historical large landowners, mostly lingayats in this case, or Tamilians who settled in the area in the 1980s. They were the first to develop irrigation to grow sugarcane, and they have accumulated sufficient capital to retain their access to water via investments in storage and micro-irrigation equipment. Today this allows them to grow highly productive long cycle crops like banana or turmeric (Table 1). They earn a high income from these crops—nearly 600,000 Rs (8,890 USD) per family worker per year—in addition to the income from the rental of the tractors they have also invested in (106,000 Rs or 1,570 USD per family worker per year, Table 2). Unfortunately, we could not estimate the income from interest on loans granted by these farmers to smallholders.

The social background of irrigating smallholders (PS3) is more diverse, from low (scheduled castes, scheduled tribes) to higher castes (lingayats). They gained access to irrigation later, in the 1990s or 2000s, generally through loans given by PS2 farmers. They cultivate turmeric associated with onion like the former category, as well as shorter cycle vegetables (with a lower productivity than the other irrigated cropping systems, see Table 1) in order to cope with the risk of groundwater depletion in borewells and price fluctuations. The yearly value added per farmer is six times smaller than for the former category (Table 2), and a portion of it serves to pay off the interest on loans taken to drill wells and purchase pumps (30% of the value added is dedicated to interest payment—Fig. 3). The yearly income per family worker—60,000 Rs (890 USD)—is hence low but remains twice as high as the poverty threshold as defined by the Indian government.

Non-irrigating smallholders in the East (PS4) are either former irrigating farmers who no longer have access to irrigation as their borewells have dried up, or farmers who never invested in irrigation. They grow sunflower or sorghum crops in Kharif season, followed by pulses in Rabi season, which have the lowest land and labour productivity (Table 1). They earn a significant additional income from livestock farming (67% of their agricultural income) and from their agricultural labour which they sell to irrigating farmers (17% of their total income), and altogether they earn an income close to the poverty threshold (Table 2).

Non-irrigating smallholders in the Western zone (PS5) have not yet invested in irrigation, which developed later in this part of the study area. The slightly more humid climate allows them to cultivate rainfed maize and marigold with better and more stable yields and marginally higher productivities than for the rainfed cropping systems in the Eastern zone (with the exception of marigold, with a labour-intensive harvest and hence low labour productivity, Table 1). Difficulties in accessing fodder and competition between activities for the use of labour force led them to abandon livestock farming. Agricultural labour wages thus play a more important role in their livelihoods (more than fifty per cent of the total income, which is also close to the poverty threshold, Table 2).

The majority of the landless labourers with livestock (PS6) have never owned land and work on other people’s farms as day labourers. Benefitting from the development of the milk collection scheme, they set up a small dairy herd which they feed on grazing land, with spontaneous collected fodder and purchased straw and concentrate. This livestock activity is labour intensive, but with the recent changes in irrigated agriculture, it is more secure than the local sale of daily labour, and also limits the need to migrate to more distant destinations such as plantations in Kerala. For these landless workers, livestock farming represents over half their total income, which is equal to the poverty threshold (Table 2).

Non irrigating farmers and landless people (PS4, PS5 and PS6) rather belong to low or middle castes, except in the East part of the area where groundwater level is critical, hence many farmers, including lingayat, went back to rainfed agriculture.

## Discussion

### Greater wealth creation, more employment and higher incomes: irrigation as a tool for reducing poverty

The impact of irrigation on poverty reduction^4,7^ is confirmed by the results obtained in this research, where it is illustrated in an original manner by including labour and its productivity in the analysis. In the study zone, under the current price conditions, irrigated cropping systems indeed produce between 6 to 20 times more value added per hectare than rainfed crops. As they require more labour (up to seven times more workdays per hectare) they are capable of creating employment. The increased demand for labour is due to the irrigation work itself but also and above all to the multiplication of crop cycles, to the increase in yields with increased time dedicated to harvest and post-harvest activities and to the importance of manual labour in vegetables cropping systems. But to create employment, the irrigated cropping systems must however permit an increase in labour productivity, which is confirmed here: the value added per workday is between 1.2 and 7 times higher for irrigated cropping systems in comparison to rainfed crops. Finally, the agricultural incomes obtained by farmers with access to irrigation are 2 to 80 times higher than those of rainfed farms. More than the increase in productivity per hectare, it is the increase in labour productivity and income that explains the farmers’ huge shift to irrigated cropping systems when they have the means. The very small size of some of the farms that have experienced such an increase must also be highlighted: 0.6 irrigated ha allows two workers to each earn an annual income of nearly 60,000 Rs (890 USD), or double the poverty threshold, a performance that can be directly attributed to irrigation. Overall, irrigation enables greater wealth creation, employment and an increase in income, particularly on very small farms and hence has been a very efficient tool to reduce poverty in Gundlupet.

### Higher dependency and greater risks

Quite rightly, irrigation is often presented as a means of reducing the sensitivity of agriculture to climate variations, of stabilising production and ensuring the availability of food for farming families between years^3^. It is nonetheless difficult to apply this reasoning to situations such as those found in Gundlupet, where the development of irrigation is accompanied by a major transformation of the cropping systems, which involve new risks.

First, irrigated crops require far higher quantities of inputs than rainfed crops. In the study area, three categories of inputs predominate: chemical fertilizers, pesticides and electricity. Irrigating farmers have become dependent on the supply of these inputs and therefore sensitive to fluctuations in their prices. Pesticide markets are little regulated and may thus vary, creating a risk for the farmers. The nitrogen-based chemical fertilizer sector is heavily subsidized in India^68,71^, generating attractive and stable prices for urea, but this is not true of other nutrients. The main subsidy irrigating farmers benefit from in the study area is undoubtedly energy, electricity with 6 h of free pumping-a-day in the state of Karnataka. This free or very low-cost access to electricity has been a major driver of the development of groundwater irrigation in India^72^. These authors estimate that if farmers were charged for power consumption at the real cost, this would represent 50 to 100% of the gross output per hectare. For these two subsidised inputs—nitrogen-based fertilizers and electricity—irrigated farms are thus highly dependent on public support, which raises the question of their sustainability.

Farmers also depend on access to output markets. With the shift from sugarcane to new irrigated crops such as vegetables, turmeric, ginger and banana, farmers using irrigation in the study area have clearly increased the risks they face in marketing their products as they have to sell them to private buyers in the market, and, unlike for sugarcane, there is no policy regulating their prices^73^. Indeed, except in the case of contract farming (as it is the case for marigold in the area), the prices of most of these new irrigated crops are highly volatile, generating sometimes large incomes, but sometimes large losses, when the selling price does not cover the production costs.

Finally, the other major risk irrigated farmers in the area face is the availability of groundwater itself. Like in other regions where the groundwater revolution occurred, the increase of groundwater pumping beyond the natural recharge capacity has depleted the groundwater^73^. In peninsular India, this difficulty is compounded by the nature of the subsoil, which makes the availability of groundwater in the deeper levels of the bedrock even more uncertain^40,74^. This generates high risks at the time of drilling new borewells—a large number are unsuccessful—but also in the use of the old ones, as the yield remains uncertain.

### Agricultural workers, historical owners, smallholders: the inequalities remain and are reinforced by certain mechanisms at work in the development of irrigation

With ratios of incomes related to agriculture ranging from one to forty in the study zone today, it is clear that inequalities persist, and irrigation has not managed to overcome them. Looking in turn at each of the different social categories and their role in irrigation development, we can identify the different mechanisms at work in Gundlupet, which on the contrary have resulted in irrigation reinforcing the existing inequalities. The first is certainly access to land, widely underscored in the literature^4,16,17^, which translates into the fact that farmers who possess more land benefit more from the productivity gains provided by irrigation.

This mechanism strongly impacts landless agricultural workers and farmers who own very little land, contributing to their marginalisation, but the increase in job availability induced by irrigation may compensate at least partly this effect. Indeed, these social categories, who are employed more frequently than before the development of borewells on landowners’ lands, have clearly benefitted from the job creation in the territory, thus helping them avoid higher levels of migration. Nonetheless, and this is a far less studied subject, the distribution of added value is extremely unfavourable to them, confirming the conclusions obtained in other irrigated regions of India^75,76^. With a daily wage of 120 Rs (1.8 USD) for women and 250 Rs (3.7 USD) for men at the time of the study, the daily wealth creation (labour productivity, Table 1) in irrigated cropping systems is two to fourteen times higher than the daily wage. Thus, although irrigation creates jobs, the salaries remain very low in comparison to the wealth created, and hence are not capable of reducing the existing inequalities. The intensity of this mechanism varies depending on the cropping systems: it is particularly high in banana cultivation that requires little labour, and less present in the case of vegetables for the opposite reason. To conclude the discussion of the characteristics of the employment induced by the development of irrigation, we should note that it is also highly sensitive to the evolution of water availability. In villages where there has been a return to rainfed agriculture, there has also been a reduction in work requirement and an increase in migration in families with little or no access to land, who were dependent on this paid labour for their survival^77^.

At the other end of the social spectrum, as highlighted in other works^20,21^, the ‘historical owners’ reap all the benefits of the development of irrigation: better-off to start with, they were the first to invest in irrigation at a time when the price of irrigated agricultural produce was high and varied little. The profits they gained allowed them to invest in different types of equipment, particularly tractors—renting out these tractors today provides them an additional income (up to 15%, Table 2)—as well as to lend money to poorer families who borrow, amongst other reasons, to sink borewells themselves. In addition to the interest they earn, these loans have allowed some owners to acquire land when the debtor was unable to meet the repayments. As underscored in other works on groundwater based irrigation^10,11,19,78^, possessing a large capital is also a clear advantage in the choice of crops, a phenomenon that is even more pronounced in the case of investors than for historical owners in Gundlupet. In comparison to the smallest irrigating farmers, this allows them to develop crops with higher labour productivity—banana, coconut, ginger—which have higher input or equipment costs. Faced with the decreasing availability of water, they can also dig more and deeper borewells, and invest in equipment that allows them to save, transport and store the increasingly precious irrigation water, hence increasing the pressure on the resource.

As for the smallholders, the “irrigation miracle” has effectively occurred for those who gained access to it and for these numerous households who were marginalised in the past, it has translated into a notable increase in their incomes (today the income of an irrigating smallholder is more than twice that of a non-irrigating smallholder). They nonetheless developed their irrigation systems in less favourable conditions than the first irrigators, both in terms of physical location (soils with a low water holding capacity), lower and far more fluctuating prices for their produce (vegetables) and lower availability of water which makes irrigated crops riskier and expensive. Above all, as they did not possess sufficient capital, they borrowed the capital required to drill their own borewells from the first irrigators. This study assesses the impact of debt, another clearly identified mechanism in rural India^79,80^: the interest on the loans taken by irrigating smallholders represents 30% of the value added created on this type of farm, hence significantly lowering their income. But what this study also shows, is that the cost of debt becomes unviable in the case of a return to rainfed agriculture as a result of the depletion of water resources in the existing borewells. The value added obtained from rainfed agriculture is indeed lower than the interest to be paid for borewells that no longer function, thrusting the households in question into critical financial situations. Shifts of this type are central to the “agrarian crisis” in India^81^ that has been widely mediatised since the 2000s, and sadly symbolised by farmers’ suicides.

### The way forward

Under these circumstances, what are the paths worth exploring to encourage a more balanced development of irrigated agriculture?

At the technical level, it is of course important to mention micro-irrigation equipment that helps save water^82^ and the spread of this equipment is already well underway for certain cropping systems in Gundlupet. When it comes to equity, the cost of this equipment represents a first limit^83^—only the largest farms having the means to invest. This cost can be counterbalanced by subsidies targeting poor populations to enable them to acquire such equipment, and this is the focus of certain development programmes in India (i.e. the Ganga Kalyana Scheme for tribal populations in Karnataka or the subsidy and power connection policies in dark-regions in Gujarat^84^. The other limit is indeed the “rebound effect” associated with micro-irrigation: at the individual level, the farmers who possess this equipment seek to use the water they save to irrigate new areas^85,86^. The pressure on water thus remains unchanged, or even increases, exacerbating the impact on unequipped irrigators, and in no way resolves the issue of equity.

Combining irrigated farming with other productive activities, less dependent on irrigation, is another path that emerges from the analysis carried out in Gundlupet. As in other parts of India, the development of milk collection has allowed numerous families to earn additional income from livestock farming and the regular sale of milk. Although most investors do not engage in livestock farming, and this activity only makes a modest contribution to the income of mid-size irrigated farms (4%), it represents over a third of the agricultural income for irrigating smallholders, two-thirds for smallholders cultivating unirrigated cereals, sunflower and pulses, and provides the landless labourers who have developed this activity an income equivalent to their earnings as day labourers. In addition, livestock farming ensures the recycling of vegetal matter (crop residues, weeds on cultivated plots) and plays a positive role in soil fertility via the application of manure^87^. Developing livestock farming nonetheless implies having the means to acquire the animals, being able to feed them and having access to the labour required to look after them. Further, although income from livestock is essential to the families in question, it nonetheless remains far below that derived from irrigated farming, confirming the results obtained in other irrigated regions in India^88^. Just like dairy farming, rainfed crops, or crops less dependent on irrigation alongside irrigated crops represent an interesting avenue to ensure a more balanced development. This is the case with marigold for example, a contract crop that requires fairly intensive labour, sunflower, which has become more widespread in the region over the last few years, or even finger millet which is becoming fashionable again amongst certain well-off urban consumers. In comparison to irrigated crops, these crops have a low labour productivity and involve certain risks (plant health and price for marigold, for example). They are nonetheless interesting in terms of diversification and a reduction in sensitivity to hazards; some also produce crop residues that can serve as animal feed and contribute to earning an income from dairy produce.

At the social level, like many other studies this analysis confirms the structural nature of the land issue in the irrigated agriculture development model and the issue of a more equal distribution of land. As we have seen, it is possible to live off 0.3 irrigated hectares per family worker, so every hectare counts! In addition to land redistribution, which has not been on any agenda in India for several decades now^89^, this could take the form of transaction regulations governing, for example, indebted farmers’ loss of their land or large irrigated farms set up by investors, a new phenomenon that impacts water resource given their level of equipment.

When it comes to managing water, in Gundlupet, like in a large part of the Indian peninsula, a great deal needs to be done to better balance the lifting of water for irrigation and the recharging of the water table to limit futile investment in borewells that do not work, and to ensure a fairer overall distribution of water between irrigators. The development of a water market is often highlighted in the literature^90^. Owing to the hard rock aquifer context of the region, with low borewell yields limiting the opportunities of sharing pumped water^74^, Gundlupet has not seen the development of such a water market, but several authors underline that it is in fact more likely to increase rather than reduce inequalities in such a context^13,17,91^. Establishing tariffs for water pumping, or, more socially and politically acceptable, providing variable power supply depending on crop needs, seems to be a more hopeful solution^92,93^. Such variable power supply could be used to encourage the development of monsoon and winter crops that have less impact on water resources, to the detriment of long cycle crops such as banana, for example, which requires a large quantity of water particularly during the dry summer period, when potential evapotranspiration is at its peak. The emergence of collective management of irrigation^94,95^ is also likely to provide answers to the water crisis and the existing inequalities in Gundlupet. This collective management can focus on the infrastructure associated with irrigation, through tubewell partnerships^69^ or in the groundwater recharge movement^96^. It can also concern the water itself, which would become a “common good” regulated by “water parliaments” assembling stakeholders at the scale of an aquifer^77^. Collective actions of this type encounter numerous obstacles but the recent national “groundwater bill” seems to have opened up possibilities in this area^97,98^.

This study also highlights a less explored field, the issue of a fairer distribution of the value added from irrigated agriculture. In the case of Gundlupet, this concerns both the interest on loans taken to buy irrigation equipment, which is a heavy burden on smallholders’ incomes, and the question of day labourers’ wages, which are far lower than the value added created. It could also involve, as is the case for investors here, value sharing between the owner, tenant and possibly the sharecropper, when the land is not exploited by the owner^75,76^. The wage daily labourers earn, which is a key issue in Gundlupet given the number of families concerned and their fragile economic situation, has almost doubled for both men and women since 2016. At the Indian level, there has been an increase in agricultural workers’ salaries over the last few years^99^. This could indicate progress in value distribution to the benefit of agricultural workers and an improvement in their livelihoods. In order to be certain this is really good news, we need to see whether this salary increase does not translate into a shift to crops that require less labour, like banana, or investment in motor-mechanised equipment for harvesting grains, for example, which would reduce job opportunities.

## Conclusion

The comparative agriculture framework used in this study, by documenting farming system dynamics and diversity in the Gundlupet Taluk, showed how the differentiation of farm trajectories over time was determined by the development of groundwater irrigation. It revealed the importance of farm initial assets—and in particular land tenure and capital—which allowed better-off farmers to seize the opportunities offered by the evolution of the global and local contexts. We showed that the development of groundwater irrigation clearly increased wealth in the region, in particular by increasing dramatically labour productivity, allowing even very small farms to generate decent livelihoods. However, this was linked with an increase of vulnerabilities, mostly related to the high price volatility of most of the irrigated crops, and to the decline in groundwater resources. We also showed that groundwater irrigation did not reduce inequality, in particular due to the high burden faced by most of the small irrigators, and, a more original result of this study, due to the unfair repartition of the value added between land owners and salaried workers.

The extensive understanding of the socio-technical functioning of the region allowed us to review and assess potential ways forward. While technological solutions to improve the efficiency of water management should be encouraged, they are unlikely to reduce both the social inequality and the pressure on the groundwater resource, and might even worsen the problem, if implemented without considering the socio-economic context. We believe that paths towards fair and sustainable development must include at least some degree of collective water management, however challenging this as proven to be, especially in India. Studies such as the one presented here, by revealing the agrarian system functioning and the farm diversity, can help taking up this challenge.

## Supplementary Information

Supplementary Information.
