# Supplementary material for: Groundwater irrigation reduces overall poverty but increases socioeconomic vulnerability in a semiarid region of southern India
Source: Sci Rep. 2022 May 25;12:8850. doi: 10.1038/s41598-022-12814-0 (PMC9132947; doi:10.1038/s41598-022-12814-0)
Supplement: Supplementary file 1 — Supplementary Information. [file 41598_2022_12814_MOESM1_ESM.pdf]

Irrigation is not a silver bullet to poverty in a semiarid region of southern India

Chloé Fischer [a,b], Claire Aubron [a], Aurélie Trouvé [b], Muddu Sekhar [c, d] and Laurent Ruiz [d, e, f]

Supplementary material - Part I

Economic assessment - adapted from Cochet, 2015a [(47)]

The gross product takes into account the agricultural produce sold as well as the agricultural production consumed by the farmer's family. The intermediate consumption are goods and services entirely consumed during the production cycle, such as seeds, fertilizers or ploughing services. Economic depreciation of fixed capital corresponds to the annual consumption of equipment and buildings of multi-year duration, such as pump, plough or tractor.

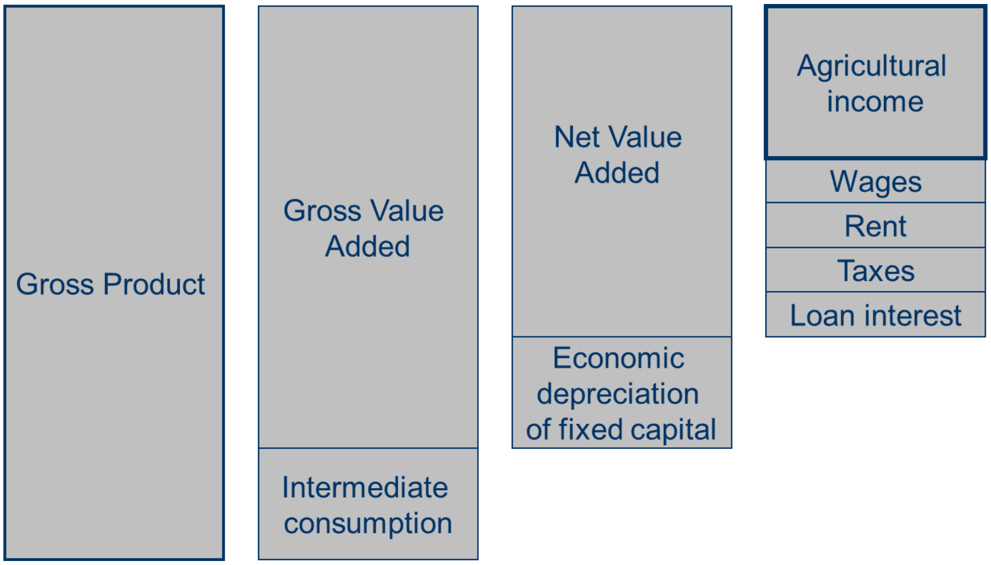

Supplementary material – Part II

Brief description of modelled cropping systems

Ginger associated with chilli	Ginger is planted during dry season (February) and harvested in December and chilli seedlings are planted in May within ginger and harvested from August to November<br><br>*Irrigation from February to December*
Vegetables and bananas on a two-year cycle	A first vegetable crop (tomato) is grown, then a second one (beetroot) is planted at the same time as the banana trees and is harvested 3 months later while banana trees are growing. Banana varieties cultivated in the area have a 11 to 15 months cycle.<br><br>*Irrigation throughout the year*
Associated turmeric	Turmeric is grown along with onions, chillis and pigeon peas. Turmeric and onions are planted together in early April, while chillis and pigeon peas are interplanted in the field one month later. Onions are harvested in early July (3 month cycle) and chilli and pigeon peas from august to November. Turmeric is harvested in January.<br><br>*Irrigation from April to December*
Vegetables (3 cycles a year)	A common succession has been chosen for the model: tomato in dry season, followed by beetroot and then cabbage.<br><br>*Irrigation throughout the year*
Sunflower followed by horsegram (East)	In Kharif season, sunflower is generally grown along with *dolichos lablab* (a variety of legumes locally called avare) and pigeon peas. After harvest, horsegram is sown during Rabi season.<br><br>*Rainfed crops*
Sorghum followed by horsegram (East)	In Kharif season, sorghum is generally grown along with pearl millet and legumes (avare, pigeon peas…). After harvest, horsegram is sown during Rabi season.<br><br>*Rainfed crops*

Maize followed by horsegram (West)	Maize is grown during Kharif season, followed by horsegram in Rabi. In the West, climate is humid enough to grow maize in Kharif that is not the case in the East.<br><br>*Rainfed crops*
Marigold followed by horsegram (West)	Marigold is sown in nursery and then planted in fields during Kharif. After harvest, horsegram is grown during Rabi season.<br><br>*Rainfed crops*

Brief description of modelled livestock farming systems

Dairy production in irrigated farms	1 dairy cow (cross-breed or holstein/Jersay type) is raised in the farm and fed with irrigated forages (Napier grass, maize), irrigated crops leftovers and weeds as well as dairy feed concentrate.
Dairy production in rainfed farms	2 dairy cows (cross-breed generally on the indigenous cattle type) are raised in the farm and fed from pasture on grazing lands (common land or around farm plot), from rainfed crop by-products (straw…) as well as dairy feed concentrate.
